# Single-center evaluation of prognostic factors for thymoma treated by surgery: a retrospective study

**DOI:** 10.1186/s13019-020-01386-7

**Published:** 2021-01-07

**Authors:** Satoshi Kamata, Itaru Ishida, Yuyo Suzuki, Hiroyuki Oura

**Affiliations:** Iwate Prefectural Central Hospital, Department of Thoracic Surgery, Ueda 1-4-1, Morioka, 020-0066 Japan

**Keywords:** Thymoma, Complete resection, Masaoka pathological stage, Postoperative recurrence, Five-year survival

## Abstract

**Background:**

This study aimed to retrospectively evaluate the clinical, pathological, and treatment-related factors associated with survival in patients with surgically treated thymomas.

**Methods:**

Sixty patients with thymomas who underwent treatment at our institution between 2004 and 2015 were included. Survival analysis was performed based on curves that were obtained using the Kaplan–Meier method. The Wilcoxon test was used for all comparisons, and *p* < 0.05 was considered statistically significant.

**Results:**

Forty-seven, four, three, four, and two patients presented tumor stages I, II, III, IVa, and IVb (according to the Masaoka classification), respectively, while six, 14, 11, 22, and seven patients had type A, AB, B1, B2, and B3 thymomas, respectively. Furthermore, 53 and eight patients underwent complete resection and required additional resection of adjacent organs, respectively, and no patients died from surgery-related complications. The five-year survival and recurrence-free survival (RFS) rates were 96 and 86%, respectively. The five-year survival rate for all stages was 100% except for those with stage IVb tumors (Masaoka classification); the survival rate for those patients was 0%. Separately, the five-year RFS rates for tumor stages I, II, III, IVa, and IVb were 100, 91, 91, 81, and 71%, respectively. Finally, the five-year survival rates in cases with complete and incomplete resections were 100 and 71%, respectively, indicating that the latter group had a significantly poorer prognosis (*p* < 0.001).

**Conclusions:**

These findings suggest that complete resection and the Masaoka pathological stage are significant predictors of prognosis in patients with thymomas. Surgery should aim to achieve complete resection; however, advanced cases may require multimodality therapy.

## Background

Thymomas are thymic epithelial neoplasms that are often treated using complete resection. However, according to the World Health Organization (WHO) classification and advanced tumors of stages III and IV of the Masaoka staging system, postoperative recurrence is observed in histologically highly malignant cases, such as types B2 and B3 thymomas. This study aimed to retrospectively evaluate the clinical, pathological, and treatment-related factors associated with survival in surgically treated thymomas.

## Methods

### Patients and ethical considerations

Among the 70 patients who underwent surgery for thymic epithelial tumors at our institution between 2004 and 2015, 60 with thymoma were included after excluding 7 with thymic carcinoma and 3 with thymic carcinoid. The study was approved by the institutional review board of our hospital. The requirement for individual patient consent was waived because only routine patient data were used to conduct this retrospective analysis.

### Study design and follow-up

The disease stage was determined using the Masaoka-Koga Stage Classification System for Thymic Epithelial Tumors, and the Tumor, Node, Metastasis Classification, eighth edition. All surgical specimens were reviewed by pathologists and categorized using the WHO classification system.

After the surgery, the patients underwent postoperative follow-up examinations, such as chest X-ray three times a year and computed tomography (CT) once a year to confirm the presence or absence of disease recurrence. Recurrence was identified from imaging reports or pathological examinations that were found during the review of case records.

### Statistical analyses

The JMP Version 10 statistical software program (SAS Institute Inc., Cary, NC, USA) was used to conduct all analyses. Survival analysis was performed based on curves obtained using the Kaplan–Meier method. The Wilcoxon test was used for all comparisons, and *p* < 0.05 was considered statistically significant.

## Results

### Patient characteristics

Table 1 shows the background information of all patients. The cohort included 30 males and 30 females with a mean age of 61 years (range: 25–82 years). Moreover, the mean duration of the postoperative follow-up period was 81 months; only deaths caused by the primary disease were considered cases of mortality in this study.

**Table 1:** Patient characteristics

Characteristics	
Age (years)	
Mean ± SD	61 ± 14.9
Range	25–83
Sex, n(%)	
Male	30(50)
Female	30(50)
Masaoka stage, n (%)	
I	47(78)
II	4(7)
III	3(5)
IVa	4(7)
IVb	2(3)
TNM stage, n(%)	
I	51(85)
II	1(2)
IIIa	2(3)
IIIb	0(0)
IVa	5(8)
IVb	1(2)
Thymoma-related syndrome, n(%	
Myasthenia gravis	11(18)
Pure Red Cell Aplasia	1(2)
Lichen planus	1(2)
Histological type, n(%)	
A	6(10)
AB	14(23)
B1	11(18)
B2	22(37)
B3	7(12)
Tumor size (cm)	
Mean ± SD	5.4 ± 2.2
Range	1.1–10
Operative approach, n(%)	
Median sternotomy	39(65)
Lateral incision	1(2)
Clamshell incision	5(8)
Video assisted thoracoscopic surgery	15(25)
Operative procedure, n(%)	
Extended thymectomy	17(28)
Thymomectomy	43(72)
Operative time (min)	
Mean ± SD	184 ± 79
Range	40–362
Blood loss (g)	
Mean ± SD	94 ± 78
Range	1–360
Postoperative treatment, n(%)	
Radiotherapy	12(20)
Chemotherapy	4(7)
Resection status, n(%)	
Complete resection	53(88)
Incomplete resection	7(12)
Initial relapse site (cases)	
Pleural dissemination	6
Pulmonary metastasis	1
Local	1
Treatment for recurrence (cases)(overlap)	
Surgery	1
Chemotherapy	
protocol	
ADOC	2
CBDCA+PTX	1
Cisplatin + Etoposide	1
Radiation	4

*ADOC* Adriamycin + cisplatin + vincristine + cyclophosphamide, *CBDCA* carboplatin, *PTX* paclitaxel, *SD* standard deviation, *TNM* tumor, node, metastasis

### Surgical treatment and perioperative period

Median sternotomy, lateral thoracotomy, the clamshell approach, and the complete thoracoscopic approach were employed in 39, one, five, and 15 patients, respectively. Additionally, 17 patients underwent extended thymectomy, while 43 underwent thymomectomy (Table 1).

Additional combined resection of adjacent organs was performed in eight patients; of these patients, six, four, one, and one underwent resection of the lung, pericardium, superior vena cava, and diaphragm, respectively. No perioperative mortality was observed, but 10 patients (16.7%) developed postoperative complications including myasthenic crisis (*n* = 2), Horner’s syndrome (*n* = 1), chylothorax (*n* = 1), respiratory failure (*n* = 2), phrenic nerve palsy (*n* = 3), and postoperative failure of the sternal closure (*n* = 1).

### Stage classification, therapeutic modalities, and patient prognoses

**Fig. 1:**
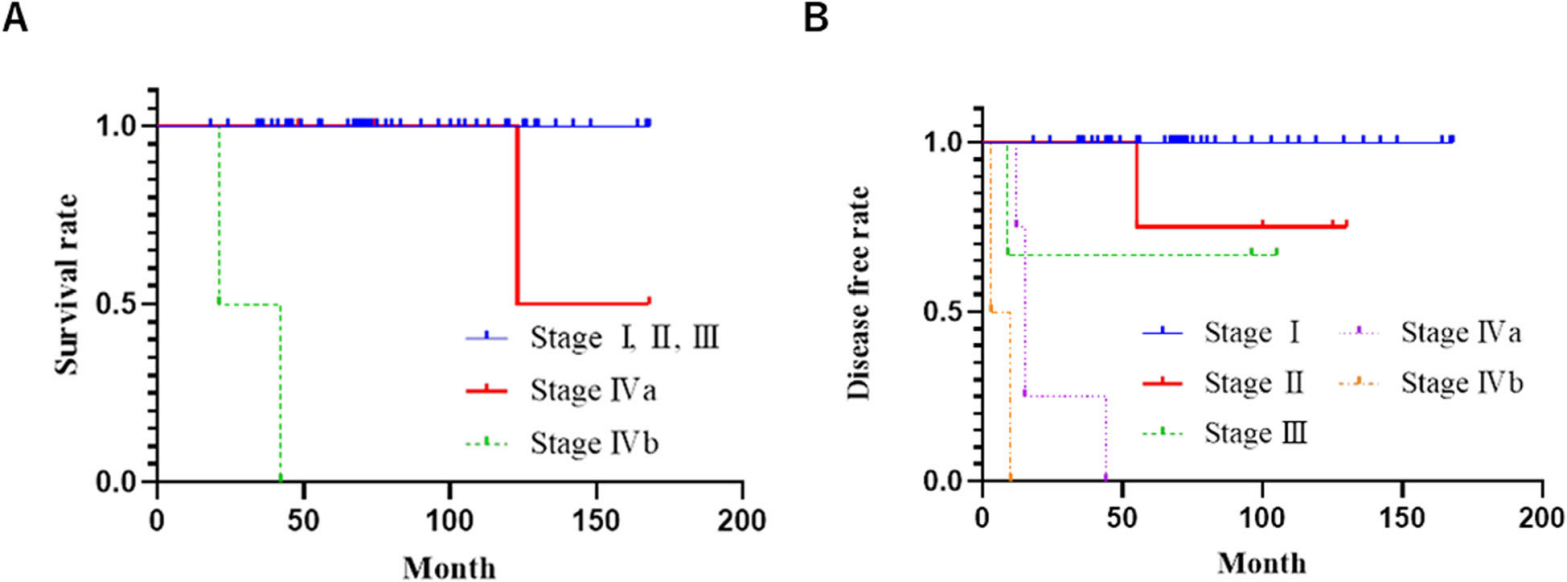
Survival and recurrence-free survival curves according to the Masaoka classification. **a** Survival curves. **b** Recurrence-free survival curves

**Fig. 2:**
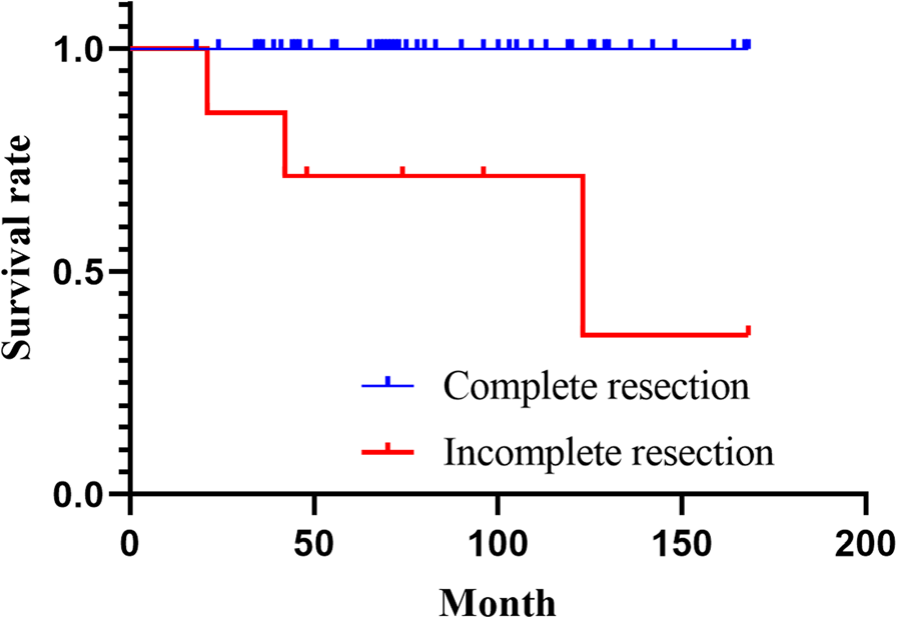
Survival curves according to the resection status

Among the 60 patients with thymomas, 47, four, three, four, and two had tumors of stages I, II, III, IVa, and IVb, respectively (Table 1). The overall five-year survival and recurrence-free survival (RFS) rates were 96 and 86%, respectively. Among patients with stage I, II, and III thymomas, no cases of mortality were observed during the follow-up period. The five-year survival rates of those with stage IVa and IVb thymomas were 100 and 0%, respectively (Fig. 1a). Although the five-year survival rate of patients with stage IVa thymomas was 100%, one of the four patients died of intrathoracic disseminated recurrence 123 months after surgery, while two patients with stage IVb tumors died of pleural disseminated recurrence 21 and 42 months after surgery, respectively. Meanwhile, the five-year RFS rates in stage I, II, III, IVa, and IVb tumors were 100, 75, 67, and 0%, respectively (Fig. 1b). Patients with stage I or II tumors and III or IV tumors demonstrated five-year RFS rates of 100 and 78%, respectively; therefore, stage III and IV thymomas were associated with a significantly poorer prognosis (*p* < 0.001) (Table 2). The five-year survival rate among the patients who underwent incomplete resection (71%) was significantly lower than that among those who underwent complete resection (100%), indicating a poorer prognosis in the latter group (*p* < 0.001) (Fig. 2).

**Table 2:** Univariate analysis of prognostic factors

Variables	***n*** = 60	Five-year survival (%)	***P***
Sex			
Male	30	93	0.225
Female	30	100	
Age (years)			
< 50	15	100	0.376
≧50	45	95	
Masaoka stage, n (%)			
I-II	51	100	< 0.001
III-IV	9	78	
Histological type			
A-B2	52	98	0.074
B3	8	86	
Myasthenia gravis			
present	11	100	0.478
absent	49	96	
Resection status			
Complete resection	53	100	< 0.001
Incomplete resection	7	71	
Recurrent-free interval (years)			
< 3	6	83	0.307
≧3	2	100

### Prognostic evaluation based on the operative procedure

**Fig. 3:**
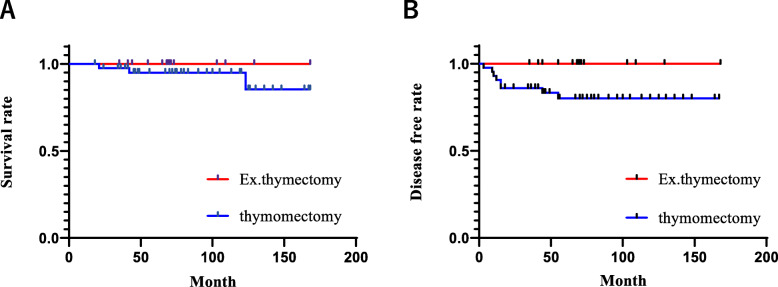
Survival and recurrence-free survival curves according to operative procedure. **a** Survival curves. **b** Recurrence-free survival curves

Prognosis and recurrence-free survival were evaluated according to the differences between the surgical methods of thymectomy and extended thymectomy. The 5-year overall survival for thymectomy and extended thymectomy were 100 and 95%, respectively, and recurrence-free survival rates were 100 and 80%, respectively. Therefore, extended thymectomy tended to be associated with better prognoses in this study (Fig. 3).

### Prognostic evaluation based on the WHO classification

**Fig. 4:**
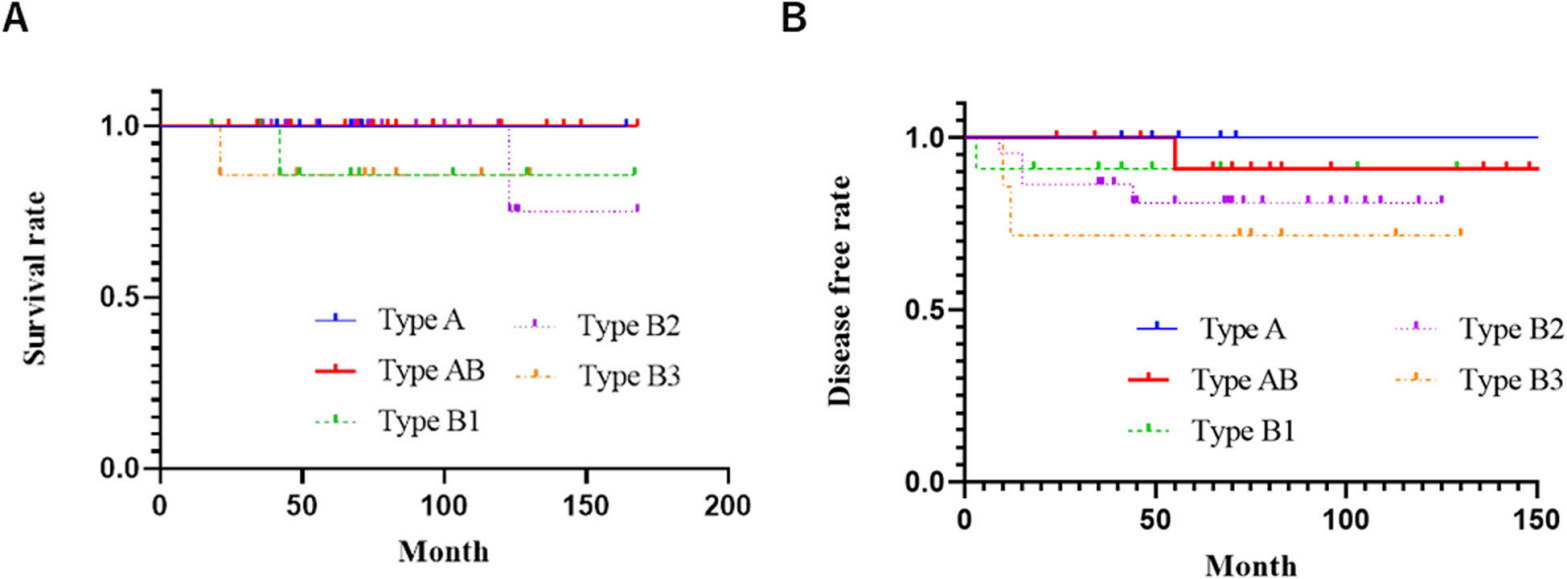
Survival and recurrence-free survival curves according to the World Health Organization classification. **a** Survival curves. **b** Recurrence-free survival curves

According to the histological type (WHO classification), the cohort included six, 14, 11, 22, and seven thymomas of types A, AB, B1, B2, and B3, respectively. Additionally, the five-year survival rates for types A, AB, B1, B2, and B3 were 100, 100, 86, 100, and 86%, respectively (Fig. 4a). The corresponding five-year RFS rates were 100, 91, 91, 81, and 71%, respectively (Fig. 4b).

### Autoimmune complications of thymomas

The autoimmune complications of thymomas included myasthenia gravis, pure red cell aplasia, and lichen planus in 10, one, and one case(s), respectively. Overall, autoimmune complications were observed in one (17%), one (7%), four (36%), seven (31%), and zero (0%) cases with types A, AB, B1, B2, and B3 tumors, respectively. The presence of autoimmune complications was significantly higher among patients with thymomas of types B1 and B2. Myasthenia gravis did not affect the prognosis of patients with thymomas (Table 2).

### Treatment for recurrence and postoperative prophylactic treatment

Overall, six, one, and one patient(s) developed pleural dissemination, local mediastinal lesions, and lung metastasis during initial recurrence, respectively; thus, pleural dissemination was the most common postoperative complication. Surgical resection for pleural dissemination was performed in one recurrent case.

The most frequently used chemotherapeutic regimen for the treatment of recurrence was adriamycin + cisplatin + vincristine + cyclophosphamide (ADOC), followed by carboplatin (CBDCA) + paclitaxel (PTX) and cisplatin + etoposide, respectively. After undergoing initial surgery for the primary lesion, three patients received prophylactic ADOC, while one received CBDCA + PTX chemotherapy.

Post-recurrence radiotherapy was administered to four cases with pleural dissemination, while three (6%), two (50%), two (67%), and five (83%) patients with stages I, II, III, and IV thymomas received postoperative prophylactic radiotherapy, respectively. One patient with stage IV thymoma refused postoperative prophylactic radiotherapy.

### Prognostic evaluation based on the recurrence-free period

**Fig. 5:**
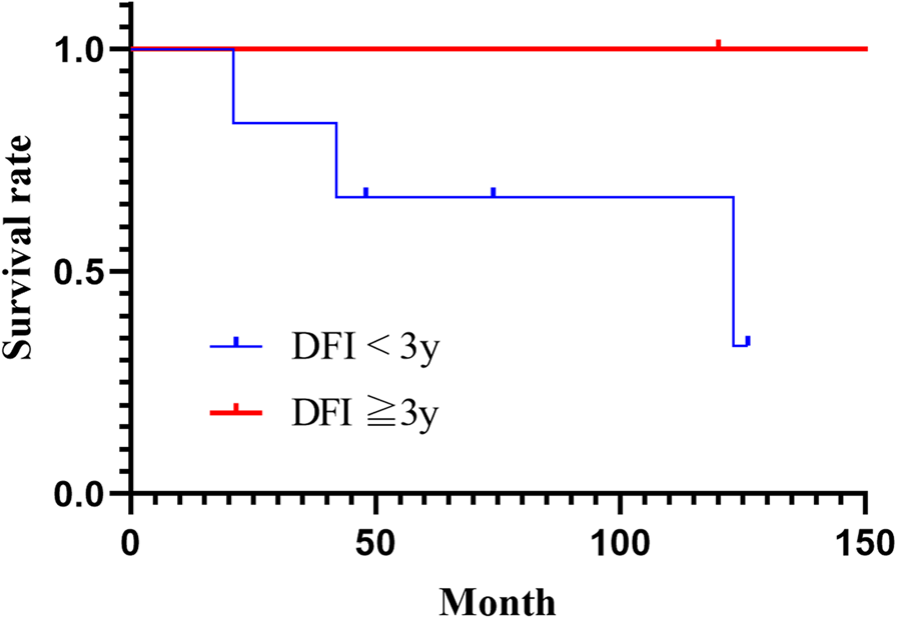
Survival curves according to disease-free intervals. DFI, disease-free interval

The recurrence-free period was less than 3 years and 3 years or more in six and two recurrent cases, respectively. The corresponding five-year RFS rates were 83 and 100%, respectively, indicating a poorer prognosis in patients with a recurrence-free period of less than 3 years, compared to 3 years or more (Table 2, Fig. 5).

## Discussion

The most effective surgical modalities or approaches for stage I and II tumors remain controversial. Total thymectomy is generally performed during the removal of the tumor; however, the removal of only the tumor or partial thymectomy during tumor removal has also been explored. Because even a small tumor can lead to intrathymic metastasis and multiple thymomas, it has been argued that total thymectomy should always be considered [1]. The roles of the thymic tissues in adults remain largely unclear; therefore, further investigations are necessary to determine they should be preserved. In the presence of autoimmune complications, such as myasthenia gravis, extended thymectomy, including the surrounding adipose tissue, should be considered. Myasthenia gravis may also develop after total thymectomy; therefore, the thymus may play a partial role in autoimmune diseases. In the present cohort, one patient who had undergone thymomectomy and who had type-B2 thymoma (according to the WHO classification) developed myasthenia gravis 2 years after surgery.

Median sternotomy has been reported as the most common surgical approach; however, thoracoscopic resection has also been adopted by many institutions in recent years. Larger tumors carry a risk of pleural dissemination, even after surgery [2]; therefore, surgical modalities should be selected carefully.

In this study, the overall survival and relapse-free survival of patients who underwent thymomectomy were not significantly different from those of the patients who underwent extended thymectomy (overall survival and relapse-free survival, *p* = 0.326 and *p* = 0.0649, respectively). Extended thymectomy tended to be associated with better prognoses. Moreover, a prior study comparing thymectomy and thymomectomy reported that the former typically presented with a higher recurrence rate, particularly local recurrence, than the latter [3]. Therefore, our results appeared to be similar to these findings.

In thymomas, the degree of malignancy varies greatly depending on the pathological condition. Type A, AB, and B1 thymomas (according to the WHO classification) are often cured by total resection, while types B2 and B3 are invasive and usually highly advanced by the time of detection, leading to significantly high rates of postoperative recurrence [4]. Among eight cases with recurrence in this study, six (75%) and seven (88%) tumors were of WHO types B2 and B3 and Masaoka stages III or IV, respectively. Separately, one patient with stage II thymoma who had undergone median sternotomy thymectomy for a tumor (diameter of 7 cm) experienced recurrence. This patient later received radiotherapy for the disseminated lesion and is surviving without any further disease recurrence 5 years after their initial relapse.

The most significant predictive factor for poor prognosis in thymoma is incomplete resection [5]. In this study, the five-year survival rate among patients with incomplete resection was 71%; the rate was 100% for those with complete resection. Therefore, a significantly poorer prognosis was observed in the former group. A previous report indicated that the duration until recurrence is an independent prognostic factor of prognosis [6], and the findings in the present study were consistent with the previous report.

Previous studies have indicated that chemotherapeutic regimens administered for postoperative recurrence are usually used concomitantly with radiotherapy and include adriamycin, cisplatin, and vincristine. Other effective agents include taxanes and etoposide [7]. The application of regimens, such as anticancer drugs with prednisolone and steroid pulse therapy, has also been considered [8]. In the present study, one patient with stage IVa, type B2 thymoma received steroid pulse therapy for a postoperative recurrence, with subsequent successful shrinkage of the recurrent lesion. Type AB, B1, and B2 thymomas contain abundant immature T-cells with certain differentiation tendencies; additionally, thymoma epithelial cells express steroid receptors [9]. Therefore, these tumors are amenable to the effects of steroids.

Tumors in stages I and II are usually completely resectable, while postoperative radiotherapy is required only in cases with incomplete resection or suspected involvement of the surgical margin. In stage III, postoperative radiotherapy is recommended in cases with a high risk of local recurrence due to incomplete resection and other factors. However, the efficacy of postoperative radiotherapy has not been verified in patients undergoing complete resection [10, 11]. Because recurrence is more common in the pleura and other distant regions outside the irradiated field than in the mediastinum, research has shown that radiotherapy does not improve survival following complete resection [12]. After incomplete resection, postoperative radiotherapy significantly reduced the frequency of intramediastinal recurrences and improved local control; however, due to the high frequency of pleural recurrences outside of the irradiated field [10], the overall survival is generally not influenced [13]. Consequently, radiotherapy applied to the mediastinum after complete resection of stage IV tumors effectively controls mediastinal recurrences, but does not alter overall survival. Therefore, the selection of therapeutic modalities should be individualized. Since postoperative radiotherapy is beneficial for controlling local recurrence, it should be considered for thymomas of stage III or higher; further evaluation is required to determine its efficacy regarding stage II tumors. Despite being performed at a single center, the present study evaluated individual cases in detail and, therefore, provides reliable data for subsequent research in the field of thymomas.

This study has several limitations, which include the fact that it was conducted at a single institution, it had a relatively small sample size, and it was a retrospective study. We aim to perform additional investigations by expanding the research to other institutions.

## Conclusions

The findings of this study show that complete resection and the Masaoka pathological stage may be significant factors for determining the prognosis of patients with thymomas. Larger, multicenter prospective studies are warranted to confirm our findings.

## Data Availability

The datasets generated and/or analyzed during the current study are not publicly available due to the use of internal records of patient data and the established privacy policy but are available from the corresponding author upon reasonable request.

## Abbreviations

ADOC: Adriamycin + cisplatin + vincristine + cyclophosphamide
CBDCA: Carboplatin
PTX: Paclitaxel
RFS: Recurrence-free survival
WHO: World Health Organization
